# Responding to mental health needs of Syrian refugees in Turkey: mhGAP training impact assessment

**DOI:** 10.1186/s13033-020-00416-0

**Published:** 2020-11-11

**Authors:** Akfer Karaoğlan Kahiloğulları, Esra Alataş, Fatmagül Ertuğrul, Altin Malaj

**Affiliations:** 1 WHO Country Office Turkey, Ankara, Turkey; 2 Ministry of Health of Turkey, Ankara, Turkey

**Keywords:** Refugee mental health, mhGAP, Turkey

## Abstract

**Background:**

About 1468 Syrian and Turkish doctors, serving in primary health care, have received the mhGAP training during 2016–2019. As additional training needs were identified, MoH and WHO wanted to understand the usefulness of the training and its impact in responding to the mental health needs of Syrian refugees in Turkey.

**Methods:**

A five component assessment was done in 2019, consisting of feedback of trainees, assessment of increase in knowledge; utilization of service; compliance to treatment guidelines and service user satisfaction. The purpose was to understand the perception of participants on the training; estimate the knowledge gained—attributable to the training; estimate the increase in mental health cases identified and treated; compliance with treatment guidelines; as well as perception of the services received by end-beneficiaries.

**Results:**

Results indicate that most of the respondents were happy with the training, but preferred additional mhGAP training as a refresher course in the future. There was knowledge gained due to the training, 9% for the Syrian and 5% for Turkish doctors. The knowledge gained has helped the practicing doctors to be more attentive and increase the numbers of cases diagnosed after the training for almost all groups of diagnoses. Most doctors, observed during practice, comply with the guidelines shared during the training, but improvement is still needed when it comes to prescription and treatment of certain conditions. The average number of mental health cases identified increased by 38 (%27) cases in the year following the training. We observed over 70% compliance with guidelines for 9 out of 12 criteria in question. The results of the patient exit interviews indicate a high level of satisfaction with the MHPSS services provided. About 95% of beneficiaries were happy with the quality of the service, and 92% having their needs met.

**Conclusions:**

The mhGAP training was found useful. More training should be conducted to fill in the gap in service provision and meet the mental needs of Syrian refugees in Turkey.

## Background

Numerous studies have highlighted the role that war, armed conflict, torture, hunger, forced migration, and post-migration assimilation to host population have in mental health illness [1, 2]. Being a refugee/migrant, suffering or witnessing violence, daily stressors, living conditions, low socioeconomic status, being a female and of young age, increases the likelihood of feeling hopeless, afraid and worried.

Ultimately, the mental health disorders (post-traumatic stress disorder; depression and anxiety) will take a toll, but especially women, children and adolescents will be more affected. Women that have suffered violence, rape, intimate partner violence are more prone to PTSD, as are children and adolescents, especially in temporary shelters [3, 4].

Literature on the prevalence of mental disorders showed higher prevalence rates for the refugee population, but the prevalence rates are heterogenous in different studies [5–8].

The burden of mental health illness is made worse by numerous barriers to service provision, linked to stigma, language, financial, legal status, social and cultural beliefs, and information sharing reasons [9, 10]. The situation is often exacerbated by restrictive migration policies, health system challenges (human and financial resources), as well as service providers barriers [11]. In addition to dealing with the disorders themselves, one need to also address the other issues related to poverty and social exclusion.

Turkey is hosting 3.6 million Syrians under temporary protection [12]. This is the largest number of refugees hosted in one country since World War II. Most of the Syrians reside in the south-eastern provinces, close to the border. Less than 2% of them are in 7 temporary shelters. Turkey has maintained an open-door policy and has offered shelter, food, education and health services, for free, to millions of Syrian refugees and others, and the United Nations Regional Refugee and Resilience Plan actors have complemented this support.

Operational data from the MoH and assessments done by WHO, since 2016, indicate that Syrians have the same burden of disease and risk factors as the host population in Turkey, for most non-communicable diseases, excluding mental health [13].

The mental health disorders of refugees fleeing from war and destruction became a priority for the Ministry of Health and WHO in Turkey. The treatment gap for mental disorders is more than 50% in general and may reach to 90% in low resource countries [14]. Traumatic events such as war and forced migration are expected to increase the treatment gap. The resulting treatment gap has been described as 89% for PTSD, 90% for anxiety, and 88% for depression [10]. The early identification and treatment remain crucial for easing the burden of mental health disorders.

Turkey has the lowest number of psychiatrists in the WHO European Region. The number of other mental health professionals and inpatient beds allocated to psychiatric care is also limited, compared with other European countries [15, 16]. Considering the limited resources in the area of mental health, integration of mental health services to primary care services is crucial for decreasing the identification and treatment gap. The Ministry of Health in Turkey has established a network of primary health centers, as part of the Turkish family physician network, that provides free primary care services, by training and hiring thousands of Syrian health professionals that were part of the refugee population in the country. It was agreed that the World Health Organization’s mental health Gap Action Program (mhGAP) would be adopted and utilized to train hundreds of Syrian and Turkish health professionals, and the services provided in the network of refugee health centres, would include both physical and mental health services.

The WHO Mental Health Gap Action Programme (mhGAP) aims at scaling up services for mental, neurological and substance use disorders, especially for countries with low- and middle-income [17]. There is evidence that mhGAP guidelines can be used to successfully train non-specialized health workers, in resource-limited settings, to recognize and treat common mental illnesses [18]. Training schedule and/or content would need to be adopted to the local context [17].

The training program, modified for Turkey, includes the following modules: introduction to mhGAP, essential care and practice, depression, psychosis, child and adolescent mental health, dementia, self-harm/suicide, and, stress related disorders. The epilepsy and substance-use disorders were covered in other MoH training programs, hence these two modules were taken out from this training. Two additional topics—promotion of mental health and anxiety disorders—were added to the program, based on feedback received from GPs. The training materials were reviewed by local experts and adopted to the country context.

MoH and WHO trained about 1,468 Syrian and Turkish doctors on mhGAP between 2016 and 2019. According to reports published so far, this is the largest number of doctors trained in mhGAP anywhere in the world.

The training teams consisted of MoH and WHO staff and consultants, working as a multidisciplinary team, from design, training of trainers, training of doctors, training evaluation and assessment.

In mid-2019, additional training needs were identified by the MoH and shared with WHO. It was jointly decided to conduct an evaluation of the training implemented so far, before additional training sessions were organized. The original hypothesis was that the training would enable Syrian and Turkish GPs to better identify, diagnose and treat mental health disorders.

## Methods

It was assumed that mhGAP training of non-specialized primary care personnel would increase the early detection and treatment of cases needing mental health and psycho-social support. This study aims to provide a comprehensive picture on how the MoH and WHO training program helped Syrian and Turkish health professionals (GPs) get more knowledge on mental health disorders, diagnosis and treatment; and how it lead to a service improvement for the Syrian refugees in need of mental health services in Turkey.

The proposed design of this mhGAP training impact assessment called for 5 components to be completed:

- The first component consisted of an online survey that collected information from training graduates on the overall impression on the training, most important topics and changes needed. The online survey was open to all mhGAP graduates, for a period of 3 months. Links to Google Forms were sent to all trained GPs by the MoH.
- The second component consisted of pre/post-tests conducted between the 1 week of mhGAP training. The pre/post-test were created using multiple choice questions selected from the general question bank of the mhGAP training manual. The comparison between posttest and pretest scores gave an idea of the information and/or knowledge transferred during the five days of training. Pre/post-test results of mhGAP trained doctors were retrieved from the MoH-WHO training datasets and analyzed using paired student’s T-test.
- The third component consisted of the analysis of the average number of cases identified by trained doctors—during 1 year before and one year after the training. The hypothesis was that the number of cases identified would increase after the training. The numbers of the mental health related diagnoses, for a period of one year before and one year after the training, were extracted by the MoH Health Information System for 200 doctors trained in mhGAP.
- The fourth component consisted of a compliance assessment: the trained doctors, who were practicing, were observed for at least one working day (preferably 5 mental health cases), on how they complied to a set of criteria from the guidelines discussed during the training. mhGAP trainers (psychiatrists) were sent to observe randomly selected doctors providing primary health care services in five provinces. The compliance of the service provision was assessed by the observer (mhGAP trainer) using a “competency assessment form” included in mhGAP training manual [17]. This form includes a set of skills categorized in 12 categories: promote respect and dignity, know common presentations, know assessment principles, know management principles, use effective communication skills, perform assessment, assess and manage physical conditions, assess and manage emergency presentations, provide psychosocial interventions, deliver pharmacological interventions as needed, follow-up, referral and link with outside agencies.

A half day training/discussion session was organized on evaluation of these categories.

- The fifth and the last component consisted of the patient exit interviews, that collected information on the perceived mental health status and satisfaction with the mental health services provided in the refugee health training centers, supported by the MoH and WHO. About 350 patients were interviewed, after receiving a MHPSS service at one of the 7 RHTCs in 7 provinces, by patient guides/data entry clerks using a standard questionnaire. Interviews were conducted by Syrian nationals or Turkish nationals fluent in Arabic.

Training of interviewers, questionnaire testing and improvement, and data collection took place during the year 2019. Data entry was done by using online forms for the perception of trainees, and patient exit interviews. Direct observation notes for each doctor were also collected at the end of each day through online forms. Data was exported to Excel 2010 and analyzed separately for each component. The data was cleaned, recoded and analyzed using Stata 16. Univariate and bivariate analysis was conducted.

The pre/post-test results were analyzed using the Student’s paired T test. The difference between the scores was reported for findings significant at the p < 0.05 level.

The data on utilization of services, measured as the number of mental health cases diagnosed one year before and compared with number of cases diagnosed one year after the training, was analyzed. The data was retrieved from the MoH database as consultations per general practitioner, broken down by age-group and gender and major diagnosis. The data was re-organized by calculating the overall number of diagnoses by using a code for each GP as unique identifier. The results were analyzed using Students’ paired T test. The statistically significant results were reported for p < 0.05.

The compliance with 12 criteria and their components were analyzed using univariate analysis and reported as percent compliance per each of the 12 criteria in question.

The patient exit interviews were analyzed as univariate analysis.

## Results

The results from the findings from each component are displayed below.

### Perception of trainees: online survey

About 211 doctors responded to the MoH call for the online survey. The results of the responses measured as percent that responded positively to the question are listed in Table 1. The invitation to the doctors that completed the mhGAP training course was send via email by the MoH, and the reported email is the only unique identifier for this component of the assessment. No other demographic or other information, apart of what is displayed in Table 1, is available.

**Table 1:** Online survey results (N = 211)

Questions	% (Yes)	% (No)
Was the training useful?	96.2	3.8
Was the format of the training useful?	95.3	4.7
Training length needs to be changed	43.1	56.9
Training method needs to be changed	7.1	92.9
Content of training modules needs to be changed	21.8	78.2
Was psycho-education content useful?	86.7	13.3
Refresher course needed?	82.9	17.1

The majority of respondents reported the training was useful (96.2%); top two most useful topics were ‘promotion of mental health’ (55.0%) and ‘depression’ (37.0%); while the least useful topic was ‘psychotic disorders’ (34.6%). The format of the training was useful for 95.3% of the respondents. Only 28.9% proposed new topics, on the following: addiction, child and elderly psychiatry, and pharmacological treatment of disorders. The psycho-education part was found useful (86.7%). About 53% responded that they have no difficulty with the implementation of psycho-education part. A refresher course was needed by 82.9% of the respondents. The online survey component confirmed that the training was useful and further training may be supported in the future. As no other socio-demographic data was available, further analysis was not possible regarding the ethnicity, age or job placement of the respondents.

### Assessment of increase in knowledge: pre/post-tests

The mhGAP training for doctors was designed in a way that the information and/or knowledge conveyed during the training would be measured by using pre-and posttests, consisting of 25 questions on topics discussed during the training itself. The time between the tests was 5 days. The results were registered as number of correct answers and converted as percent correct. Student’s paired T test was used to estimate whether there was measurable improvement in score during the post-test and whether the improvement could be attributed to the training. The results of 388 Turkish doctors’ pre/posttest results are depicted in Table 2.

**Table 2:** Results of the Student’s T test (Turkish doctors, N = 388)

Variable	Obs	Mean	Std. err	Std. dev	[95% Conf	Interval]
Pre-test	388	81.70	0.54	10.60609	80.64239	82.75967
Post-test	388	86.86	0.56	11.03856	85.75386	87.95748
Difference	388	5.16	0.72	14.18617	3.738657	6.570621

On average, there was a 5% increase in the correct answers after the Turkish doctors training. The results are statistically significant p < 0.000 and t = 7.1573 (degrees of freedom = 387). The similar information for Syrian doctors is shown in Table 3.

**Table 3:** Results of the Student’s T test (Syrian doctors, N = 206)

Variable	Obs	Mean	Std. err	Std. dev	[95% Conf	Interval]
Pre-test	207	40.08	1.33	19.13046	37.45581	42.69878
Post-test	207	48.70	1.56	22.50651	45.61154	51.77976
Difference	207	8.62	0.672	9.585803	7.304796	9.931919

The Syrian doctors training resulted in a 9% statistically significant (p < 0.000) increase; t = 12.9354 (df = 206).

### Health utilization: cases diagnosed

We observed increased number of cases for all diagnoses except for the suicide/self-harm. For all the other diagnoses, we observed increased number of cases after the mhGAP training (Table 4).

**Table 4:** Comparing number of cases diagnosed before and after the mhGAP training

Variable		Before (n = 188)	After (n = 188)	t value	Prob
All cases	M SD	139.2 (9.9)	177.02 (12.2)	9.59	0.000
CAD behav and emoti	M SD	38.5 (6.9)	43.2 (8.9)	1.71	0.044
CD developmental	M SD	3.27 (0.6)	6.17 (0.9)	5.58	0.000
Dementia	M SD	7.55 (0.6)	10.77 (0.9)	7.15	0.000
Depression	M SD	75.18 (7.4)	96.41 (9.3)	6.23	0.000
Acute stress disorder	M SD	0.45 (0.1)	0.68 (0.2)	2.05	0.021
Self-harm	M SD	0.07 (0.0)	0.06 (0.0)	-0.24	0.595
Psychosis	M SD	4.25 (0.3)	5.80 (0.5)	5.69	0.000
Bipolar mood disorder	M SD	9.11 (0.7)	12.8 (0.9)	9.71	0.000
Delirium	M SD	0.80 (0.1)	1.06 (0.2)	2.12	0.018

### Compliance to treatment guidelines: direct observation

The compliance with the 12 competencies within the learning objectives of the training is shown in Table 5. For each competency, the number of components (a total of 45) is assessed and the compliance is reported as percentage. The mhGAP trainer followed doctors in 5 provinces for a total of 144 consultations and completed an assessment report. The top three competences *adhered* to were ‘promoting respect and dignity’, ‘effective communication skills’ and; ‘knowing common presentation of disorders’. The least compliant were the ‘management of these presentations’ and the ‘pharmacological interventions’.

**Table 5:** Compliance with guidelines (N = 144)

Category	Achieved %	Needs work %
Promote respect and dignity	84.0	16.0
Know common presentations	78.3	11.7
Know assessment principles	76.2	13.8
Know management principles	74.8	15.2
Use effective communication skills	81.4	18.6
Perform assessment	75.5	14.5
Assess and manage physical conditions	76.7	13.3
Assess and manage emergency presentations	40.8	59.2
Provide psycho-social interventions	67.6	32.4
Deliver pharmacological interventions as needed and appropriate	43.8	56.2
Plan and perform follow-up	67.4	32.6
Refer to specialist and link with outside agencies	72.2	27.8

### Service user satisfaction: patient exit interviews

The patient exit interviews were conducted by patient guides/data entry clerks trained by MoH and WHO, that were familiar with the facilities, questions asked, and the general sensitivity of the survey. The data was entered using Google forms.

Most of the service users interviewed were women (77.3%). The median age of the respondents was 32 (min 15- max 70). Majority of respondents had primary or secondary school training (71.2%) while 17.8% had no education. Only 11.1% percent had a university training. The majority of respondents were unemployed (72.7%). 85.4% were Arabs, 9.5% were Kurdish, and the remaining were from other ethnic background. Almost all of the respondents were Muslims, only 0.6% were Christians. 75.9% were married, while 10.4% were single and 13.7% were divorced or widowed. The majority were registered and benefited from the temporary protection in Turkey, that allowed them free services and medications. Most of them had visited a health facility for a MHPSS reason.

The results related to the type of service rendered are displayed in Table 6.

**Table 6:** Patient exit interviews, characteristics (in percentage) by gender

Characteristic	Category	Male (N = 81)	Female (N = 276)	Total (N = 357)
What service received?	Consultation	71.7	65.3	66.8
	Prescription	0.0	1.6	1.2
	Both	28.3	33.2	32.0
Satisfied with the confidentiality of service?	No	1.7	1.0	1.2
	Yes	98.3	99.0	98.8
Satisfied with relationship to staff?	No	0.0	1.6	1.2
	Yes	100.0	98.5	98.8
Satisfied with availability of service?	No	0.0	1.6	1.2
	Yes	100.0	98.5	98.8
Satisfied with opening hours?	No	8.3	3.1	4.4
	Yes	91.7	96.9	95.7
Satisfied with the length of appointment?	No	3.3	2.1	2.4
	Yes	96.7	97.9	97.6
Satisfied with the frequency of the appointments?	No	3.3	2.6	2.8
	Yes	96.7	97.4	97.2
Satisfied with waiting time?	No	1.7	5.2	4.4
	Yes	98.3	94.8	95.6
Satisfied with the information on your health?	No	0.0	2.1	1.6
	Yes	100.0	97.9	98.4
Satisfied with the information on your treatment?	No	0.0	4.2	3.2
	Yes	100.0	95.9	96.8
Would you come back for same service?	No	0.0	4.2	3.2
	Yes	100.0	95.9	96.8
Would you refer here a family member?	No	3.3	1.0	1.6
	Yes	96.7	99.0	98.4
Satisfied with quality of service	No	3.7	5.4	5.0
	Yes	96.3	94.6	95.0
Your needs were met today?	No	3.7	8.3	7.3
	Yes	96.3	91.7	92.7

The majority reported their needs were met and that they were happy with the services. The majority were also happy with all aspects of the service provided, and would either come back or refer family members for a similar service.

## Discussion

The linkage between the refugee trauma related factors and resulting mental health disorders has been properly documented [2, 19]. The interagency guidelines for MHPSS and the importance of the WHO mhGAP have been agreed, and subsequently used by the MoH in Turkey to train Syrian and Turkish doctors to handle the associated burden of mental health disorders of the refugees. A training effort continued from 2016 to 2019 and resulted in hundreds of doctors completing the mhGAP modules, as part of the adaptation training.

We conducted this multi-component training impact assessment before embarking on additional training activities. The components were conducted as separate and standalone, but the combination of the findings helped guide the decision on future trainings and improvements needed.

The first component, based on graduates’ feedback, indicated that over 96% of respondents regarded the training as useful, and 82% suggested future refresher courses on the same mhGAP topics. The most useful topics included those on promotion of mental health and depression. Depression remained an important disorder and a burden to both refugees and the service provision activities. However, the modules on psychotic disorders and self-harm were not regarded as very useful. This may be related to health services planning. MoH has suggested primary care health staff to refer patients with psychosis to the specialized mental health services. According to referral algorithms, prepared by MoH, suicide cases should be referred to the emergency services or psychiatry clinics after a first assessment. About 30% of respondents suggested to add new topics, mostly focusing on addiction; child and elderly psychiatry, and pharmaceutical treatment.

The second component, the pre-and post-tests, measured the information and knowledge transferred as part of the training. The results were similar to the other studies in the literature [18, 20]. We observed a slight knowledge increase for both Syrian and Turkish doctors. The increase was slightly bigger for the Syrian doctors, but no measurable reasons can be identified on this difference. Despite the differences in increase in knowledge attributable to the training, both Syrian and Turkish doctors benefited from the training. The question that remains is how much should the increase in knowledge attributable to the training be, so that the resources put to the training may be justified? Our suggestion was to look at how the knowledge increase influenced the improvement of the quality of services provided.

The third component, utilization of services indicated that there was a change in behavior from practicing doctors who were more attentive and identified, on average, 38 more cases of mental health cases annually, after the training. Unfortunately, the increase was not true for young ages (under 19 years old) and for the self-harm group. This highlighted that more needs to be done to address the needs of that specific age-group and type of problem. This finding was consistent with the online survey that suggested adding new training topics on child psychiatry. Discussions are underway to improve the childhood and adolescent focus in the training.

The fourth component, on the compliance with guidelines, and measuring the quality of service provided, indicated that out of the 12 criteria/guidelines, there was 80–85% compliance on 3 of them; 70–80% compliance on 6 of them, and the compliance rate was 44–68% for the remaining 3. The guidelines with the poorest compliance were those on pharmaceutical interventions and assessing and managing of disease presentations. This was in line with suggestions from the online survey indicating the need to put additional training effort on pharmaceutical treatment. Improvement needed in the pharmaceutical treatment were linked to recent changes in the legislation regulating prescription of psychiatric medications for refugees.

The fifth and final component, the patient exit interviews provided valuable information on self-reported perception of end-beneficiaries, about the MHPSS services they received. All the efforts put into training, improving the volume and quality of mental health operations, compliance with rules and guidelines, had one final objective: improving the mental health status of the Syrian refugees in Turkey. Over 95% of respondents were happy with the quality of services provided at the health facilities surveyed in 5 provinces. About 93% reported their needs met and satisfied with confidentiality, staff behavior, frequency and length of appointment. The average waiting time was 11 min. About 97% would come back for the same service and 98% would refer a family member for the same service. The results indicated that MHPSS services provided in the facilities surveyed have improved the mental health of the Syrian refugees. However, with the data we have, it is not possible to estimate the satisfaction threshold of the refugees when it comes to mental health status and services. The MoH is planning to provide additional training of trainers to another 500 doctors in Turkey, acknowledging the still existing gap in service provision.

### Limitations

The authors are aware of the limitations of the study. The number of doctors that responded to the survey is less than half of Turkish doctors trained in 2018–2019. The respondents were not selected as a representative sample, or when they completed the modules. Additionally, there might also have been a recall bias.

Although we analyzed most of the results of graduates from the recent cohorts, me missed the results of earlier cohorts. As such it is difficult to understand the differences in knowledge gained from earlier cohorts. Another limitation was the lack of a control group. However, by the time the components was completed, all the practicing Syrian doctors were already trained, as well as 900 Turkish doctors involved in service provision to Syrian refugees.

We could only include 200 practicing doctors in service utilization component due to several reasons including time of the study. Other cohorts cannot be included because either completion time of the training is less than one year or data for the utilization of services could not be retrieved from the MoH database (refugee health centres were not included to the primary care database). As such, the findings would not necessarily represent all the practicing doctors. However, the 200 sample consisted of half of the Turkish doctors trained during 2018–2019.

The compliance was estimated based on 45 parameters, converted into 12 categories. The observers were past trainers and their presence in the health facility was explained as understanding gaps and future needs for the mhGAP programme. The doctors being observed might have changed their behavior in the presence of their former trainers (Hawthorne effect).

The selection of patients for the exit interviews was not randomized. Our interviewers located in 7 refugee health training centers for a period of 30 days and interviewed patients as they exited the doctor or PSS offices. The interviews were conducted at the health facility and in confidential spaces, but it was impossible to know the extend the location of the interview might have influenced the responses of the Syrian refugees.

This assessment made use of 5 standalone components. As such different respondents contributed to the data and results of each component. The selection of respondents was not randomized. The responses came from recent cohort graduates for the online survey and pre/posttests. It was not possible to link the results from the four first components to individual doctors. However, the numbers of respondents included in the assessment helped create an understanding of the results. For future assessments, we might try to use the same training cohort and follow them during practice and include their end-beneficiaries in the study.

## Conclusions

This assessment generated evidence that supports the conclusion that the mhGAP training had a positive feedback from practicing Syrian and Turkish doctors. Knowledge was increased, that is attributable to this training, as well as a behavioral change took place, resulting in a higher number of mental health cases being identified and treated, mostly in compliance with mhGAP guidelines. Most beneficiaries responded that they were happy with the quality of services provided and that they would use the services again. Further studies are needed to understand the magnitude of the services provided and resources used to fill the gap for mental health services in Turkey.

The training was delivered by a team of peer trainers (GPs) and psychiatrists. All trainers completed a 5 days ToT training, in addition to the mhGAP training. At least 3 trainers per 25 participants were present in each session for supporting role-plays and other group activities.

## Data Availability

The data that support the findings of this study are available from Ministry of Health of Turkey but restrictions apply to the availability of these data, which were used under license for the current study, and so are not publicly available. Data are however available from the authors upon reasonable request and with permission of Ministry of Health of Turkey.

## Appendix 1: mhGAP training outline

**Table:**

Outline for mhGAP trainings	
Promotion of mental health	2 h
Essential care and practice	3.5 h
Depression	3 h
Psychosis	3 h
Anxiety disorders and stress related disorders	3 h
Suicide	2 h
Dementia	3 h
Child and adolescent mental and behavioral disorders	3 h
Other significant mental health complaints	3 h
Total duration (without breaks)	25.5 h
